# Supplementary material for: A fault-intrusion-tolerant system and deadline-aware algorithm for scheduling scientific workflow in the cloud
Source: PeerJ Comput Sci. 2021 Nov 2;7:e747. doi: 10.7717/peerj-cs.747 (PMC8576554; doi:10.7717/peerj-cs.747)
Supplement: Supplemental Information 6 [file peerj-cs-07-747-s006.zip › Myproject1.1/docs/org/cloudbus/cloudsim/examples/power/RunnerAbstract.html]

RunnerAbstract


JavaScript is disabled on your browser.


- Overview
- Package
- Class
- Use
- Tree
- Deprecated
- Index
- Help

- Prev Class
- Next Class

- Frames
- No Frames

- All Classes

- Summary:
- Nested |
- Field |
- Constr |
- Method

- Detail:
- Field |
- Constr |
- Method


org.cloudbus.cloudsim.examples.power

## Class RunnerAbstract

- java.lang.Object
- - org.cloudbus.cloudsim.examples.power.RunnerAbstract

- Direct Known Subclasses:
  :   PlanetLabRunner, RandomRunner

  ---

    

  ```
  public abstract class RunnerAbstract
  extends java.lang.Object
  ```

  The Class RunnerAbstract.
  If you are using any algorithms, policies or workload included in the power package, please cite
  the following paper:
  Anton Beloglazov, and Rajkumar Buyya, "Optimal Online Deterministic Algorithms and Adaptive
  Heuristics for Energy and Performance Efficient Dynamic Consolidation of Virtual Machines in
  Cloud Data Centers", Concurrency and Computation: Practice and Experience (CCPE), Volume 24,
  Issue 13, Pages: 1397-1420, John Wiley & Sons, Ltd, New York, USA, 2012

- - ### Field Summary

    Fields

    | Modifier and Type | Field and Description |
    | --- | --- |
    | `protected static DatacenterBroker` | `broker` The broker. |
    | `protected static java.util.List<Cloudlet>` | `cloudletList` The cloudlet list. |
    | `protected static java.util.List<PowerHost>` | `hostList` The host list. |
    | `protected static java.util.List<Vm>` | `vmList` The vm list. |
  - ### Constructor Summary

    Constructors

    Constructor and Description

    `RunnerAbstract(boolean enableOutput,
    boolean outputToFile,
    java.lang.String inputFolder,
    java.lang.String outputFolder,
    java.lang.String workload,
    java.lang.String vmAllocationPolicy,
    java.lang.String vmSelectionPolicy,
    java.lang.String parameter)`

    Run.
  - ### Method Summary

    Methods

    | Modifier and Type | Method and Description |
    | --- | --- |
    | `protected java.lang.String` | `getExperimentName(java.lang.String... args)` Gets the experiment name. |
    | `protected VmAllocationPolicy` | `getVmAllocationPolicy(java.lang.String vmAllocationPolicyName, java.lang.String vmSelectionPolicyName, java.lang.String parameterName)` Gets the vm allocation policy. |
    | `protected PowerVmSelectionPolicy` | `getVmSelectionPolicy(java.lang.String vmSelectionPolicyName)` Gets the vm selection policy. |
    | `protected abstract void` | `init(java.lang.String inputFolder)` Inits the simulation. |
    | `protected void` | `initLogOutput(boolean enableOutput, boolean outputToFile, java.lang.String outputFolder, java.lang.String workload, java.lang.String vmAllocationPolicy, java.lang.String vmSelectionPolicy, java.lang.String parameter)` Inits the log output. |
    | `boolean` | `isEnableOutput()` Checks if is enable output. |
    | `void` | `setEnableOutput(boolean enableOutput)` Sets the enable output. |
    | `protected void` | `start(java.lang.String experimentName, java.lang.String outputFolder, VmAllocationPolicy vmAllocationPolicy)` Starts the simulation. |

    - ### Methods inherited from class java.lang.Object

      `clone, equals, finalize, getClass, hashCode, notify, notifyAll, toString, wait, wait, wait`

- - ### Field Detail


    - #### broker

      ```
      protected static DatacenterBroker broker
      ```

      The broker.


    - #### cloudletList

      ```
      protected static java.util.List<Cloudlet> cloudletList
      ```

      The cloudlet list.


    - #### vmList

      ```
      protected static java.util.List<Vm> vmList
      ```

      The vm list.


    - #### hostList

      ```
      protected static java.util.List<PowerHost> hostList
      ```

      The host list.
  - ### Constructor Detail


    - #### RunnerAbstract

      ```
      public RunnerAbstract(boolean enableOutput,
                    boolean outputToFile,
                    java.lang.String inputFolder,
                    java.lang.String outputFolder,
                    java.lang.String workload,
                    java.lang.String vmAllocationPolicy,
                    java.lang.String vmSelectionPolicy,
                    java.lang.String parameter)
      ```

      Run.

      Parameters:
      :   `enableOutput` - the enable output
      :   `outputToFile` - the output to file
      :   `inputFolder` - the input folder
      :   `outputFolder` - the output folder
      :   `workload` - the workload
      :   `vmAllocationPolicy` - the vm allocation policy
      :   `vmSelectionPolicy` - the vm selection policy
      :   `parameter` - the parameter
  - ### Method Detail


    - #### initLogOutput

      ```
      protected void initLogOutput(boolean enableOutput,
                       boolean outputToFile,
                       java.lang.String outputFolder,
                       java.lang.String workload,
                       java.lang.String vmAllocationPolicy,
                       java.lang.String vmSelectionPolicy,
                       java.lang.String parameter)
                            throws java.io.IOException,
                                   java.io.FileNotFoundException
      ```

      Inits the log output.

      Parameters:
      :   `enableOutput` - the enable output
      :   `outputToFile` - the output to file
      :   `outputFolder` - the output folder
      :   `workload` - the workload
      :   `vmAllocationPolicy` - the vm allocation policy
      :   `vmSelectionPolicy` - the vm selection policy
      :   `parameter` - the parameter

      Throws:
      :   `java.io.IOException` - Signals that an I/O exception has occurred.
      :   `java.io.FileNotFoundException` - the file not found exception


    - #### init

      ```
      protected abstract void init(java.lang.String inputFolder)
      ```

      Inits the simulation.

      Parameters:
      :   `inputFolder` - the input folder


    - #### start

      ```
      protected void start(java.lang.String experimentName,
               java.lang.String outputFolder,
               VmAllocationPolicy vmAllocationPolicy)
      ```

      Starts the simulation.

      Parameters:
      :   `experimentName` - the experiment name
      :   `outputFolder` - the output folder
      :   `vmAllocationPolicy` - the vm allocation policy


    - #### getExperimentName

      ```
      protected java.lang.String getExperimentName(java.lang.String... args)
      ```

      Gets the experiment name.

      Parameters:
      :   `args` - the args

      Returns:
      :   the experiment name


    - #### getVmAllocationPolicy

      ```
      protected VmAllocationPolicy getVmAllocationPolicy(java.lang.String vmAllocationPolicyName,
                                             java.lang.String vmSelectionPolicyName,
                                             java.lang.String parameterName)
      ```

      Gets the vm allocation policy.

      Parameters:
      :   `vmAllocationPolicyName` - the vm allocation policy name
      :   `vmSelectionPolicyName` - the vm selection policy name
      :   `parameterName` - the parameter name

      Returns:
      :   the vm allocation policy


    - #### getVmSelectionPolicy

      ```
      protected PowerVmSelectionPolicy getVmSelectionPolicy(java.lang.String vmSelectionPolicyName)
      ```

      Gets the vm selection policy.

      Parameters:
      :   `vmSelectionPolicyName` - the vm selection policy name

      Returns:
      :   the vm selection policy


    - #### setEnableOutput

      ```
      public void setEnableOutput(boolean enableOutput)
      ```

      Sets the enable output.

      Parameters:
      :   `enableOutput` - the new enable output


    - #### isEnableOutput

      ```
      public boolean isEnableOutput()
      ```

      Checks if is enable output.

      Returns:
      :   true, if is enable output


- Overview
- Package
- Class
- Use
- Tree
- Deprecated
- Index
- Help

- Prev Class
- Next Class

- Frames
- No Frames

- All Classes

- Summary:
- Nested |
- Field |
- Constr |
- Method

- Detail:
- Field |
- Constr |
- Method
